# Variation of the seed endophytic bacteria among plant populations and their plant growth‐promoting activities in a wild mustard plant species, *Capsella bursa‐pastoris*

**DOI:** 10.1002/ece3.8683

**Published:** 2022-03-07

**Authors:** Byungwook Choi, Seorin Jeong, Eunsuk Kim

**Affiliations:** ^1^ 65419 School of Earth Sciences and Environmental Engineering Gwangju Institute of Science and Technology Gwangju South Korea

**Keywords:** bacterial community, cultivable bacteria, natural variation, plant growth‐promoting traits, seedling growth, weed ecology

## Abstract

Recent studies have revealed that some bacteria can inhabit plant seeds, and they are likely founders of the bacterial community in the rhizosphere of or inside plants at the early developmental stage. Given that the seedling establishment is a critical fitness component of weedy plant species, the effects of seed endophytic bacteria (SEB) on the seedling performance are of particular interest in weed ecology. Here, we characterized the SEB in natural populations of *Capsella bursa*‐*pastoris*, a model species of weed ecology. The composition of endophytic bacterial community was evaluated using deep sequencing of a 16S rDNA gene fragment. Additionally, we isolated bacterial strains from seeds and examined their plant growth‐promoting traits. Actinobacteria, Firmicutes, Alpha‐, and Gammaproteobacteria were major bacterial phyla inside seeds. *C*. *bursa*‐*pastoris* natural populations exhibited variable seed microbiome such that the proportion of Actinobacteria and Alphaproteobacteria differed among populations, and 60 out of 82 OTUs occurred only in a single population. Thirteen cultivable bacterial species in six genera (*Bacillus*, *Rhodococcus*, *Streptomyces*, *Staphylococcus*, *Paenibacillus*, *Pseudomonas*) were isolated, and none of them except *Staphylococcus haemolyticus* were previously reported as seed endophytes. Eight isolates exhibited plant growth‐promoting traits like phosphate solubilization activity, indole‐3‐acetic acid, or siderophore production. Despite the differences in the bacterial communities among plant populations, at least one isolated strain from each population stimulated shoot growth of either *C*. *bursa*‐*pastoris* or its close relative *A*. *thaliana* when grown with plants in the same media. These results suggest that a weedy plant species, *C*. *bursa*‐*pastoris*, contains bacterial endophytes inside their seeds, stimulating seedling growth and thereby potentially affecting seedling establishment.

## Graphical abstract

The interaction between plants and endophytic microorganisms has recently become a rising issue in plant ecology and evolution. We conducted both culture‐dependent and independent methods to characterize seed endophytic bacteria in the natural populations of *Capsella bursa*‐*pastoris*, a model plant species of weed ecology.

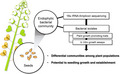

## 1 INTRODUCTION

Successful establishment of seedlings in novel environments is a major fitness component of weedy plant species and a critical factor determining species’ geographic range (Crawley, 1997). Soil microbiota, especially microorganisms in plants’ rhizosphere, is proposed to form a mutualistic interaction with plants and consequently facilitate weed establishment (Coats & Rumpho, 2014; Trognitz et al., 2016). Notably, plant seeds harbor diverse seed endophytic bacteria (SEB) (Truyens et al., 2015), so seeds and SEB of weed plants would disperse simultaneously. Since SEB would constitute microbiota inside or in the rhizosphere of seedlings (Kaga et al., 2009; Puente et al., 2009), SEB, in addition to soil microorganisms, likely influence seedling establishment in novel environments.

Despite their plausible ecological significance (Elmore et al., 2019; Jeong et al., 2021; White et al., 2018), relatively little information is available on the characteristics of SEB in weed plant species. Most information on the SEB has come from studies using agriculturally important crop plants (Card et al., 2015; Pal et al., 2019; Shahzad et al., 2018). It should be noted that crop and wild plant species tend to have distinctive characteristics. Many crop plant species have a limited number of genotypes due to strong artificial selection, and their seeds are intensively managed to have consistent quality with pathogen‐free status (Pérez‐Jaramillo et al., 2016; White et al., 2019). Consequently, SEB of crop species are suggested to have lower diversity than wild plant species. Studies on the indigenous SEB of wild plant species are required to evaluate the ecological significance of SEB (Wassermann et al., 2019).

This study examined the bacterial community of seeds in one of the most common weedy species, *Capsella bursa*‐*pastoris* (Brassicaceae) (Neuffer, 2011). *C*. *bursa*‐*pastoris* is an annual weedy plant species worldwide, except for regions near the equator (Neuffer, 2011). As a model system of weed ecology, the adaptive divergence of *C*. *bursa*‐*pastoris* along environmental gradients like altitude and latitude has been well documented (Huang et al., 2012; Neuffer, 2011).

Studies testing seed endophytes have often used DNA fingerprinting methods such as polymerase chain reaction‐denaturing gradient gel electrophoresis (PCR‐DGGE) or next‐generation sequencing (NGS) (Beckers et al., 2016; Liu et al., 2019; Xu et al., 2014). While such techniques have the advantage of detecting uncultivable bacteria, they provide scant information on the functional role of microorganisms in plant performance. In contrast, culture‐dependent methods can characterize the biological functions of isolated bacteria, although cultivable bacteria are only a subset of the whole microbiome. In particular, diverse assays can be conducted to evaluate whether SEB are able to produce so‐called plant growth‐promoting (PGP) molecules like indole‐3‐acetic acids (IAA), siderophores, 1‐aminocyclopropane‐1‐carboxylic acid (ACC) deaminases, and phosphate‐solubilizing molecules (Santoyo et al., 2016; Shahzad et al., 2018). Since culture‐independent and ‐dependent methods provide complementary information on the SEB, this study used both methods.

Previous studies using crop plants suggested that the composition and function of SEB likely depend on cultivar genotypes and growing environments (Hardoim et al., 2012; Xu et al., 2014). Weedy plants occur in a broad geographic range with diverse environmental conditions. Distinctive genotypes tend to constitute natural populations in different environments (Linhart & Grant, 1996; Neuffer, 2011), implying that the composition and function of SEB in weedy species may be population specific. Thus, for a more general conclusion on the ecological role of SEB in weedy species, a comparative study at the population level was conducted.

Here, we collected seeds of *C*. *bursa*‐*pastoris* populations along a latitudinal gradient in South Korea and characterized their SEB using both culture‐independent and culture‐dependent methods. NGS technique was used to compare SEB community among *C bursa*‐*pastoris* natural populations. In addition, SEB were isolated from seeds, and their PGP activities were assessed by examining the production of PGP molecules and the effect of SEB on seedling growth. Specifically, we addressed the following questions: (i) What kinds of endophytic bacteria inhabit inside seeds of *C*. *bursa*‐*pastoris*? (ii) Do *C*. *bursa*‐*pastoris* natural plant populations have differential seed bacterial communities? (iii) Do populations contain SEB‐exhibiting PGP traits and promote seedling growth?

## 2 MATERIALS AND METHODS

### 2.1 Study populations and seed sources

**FIGURE 1:**
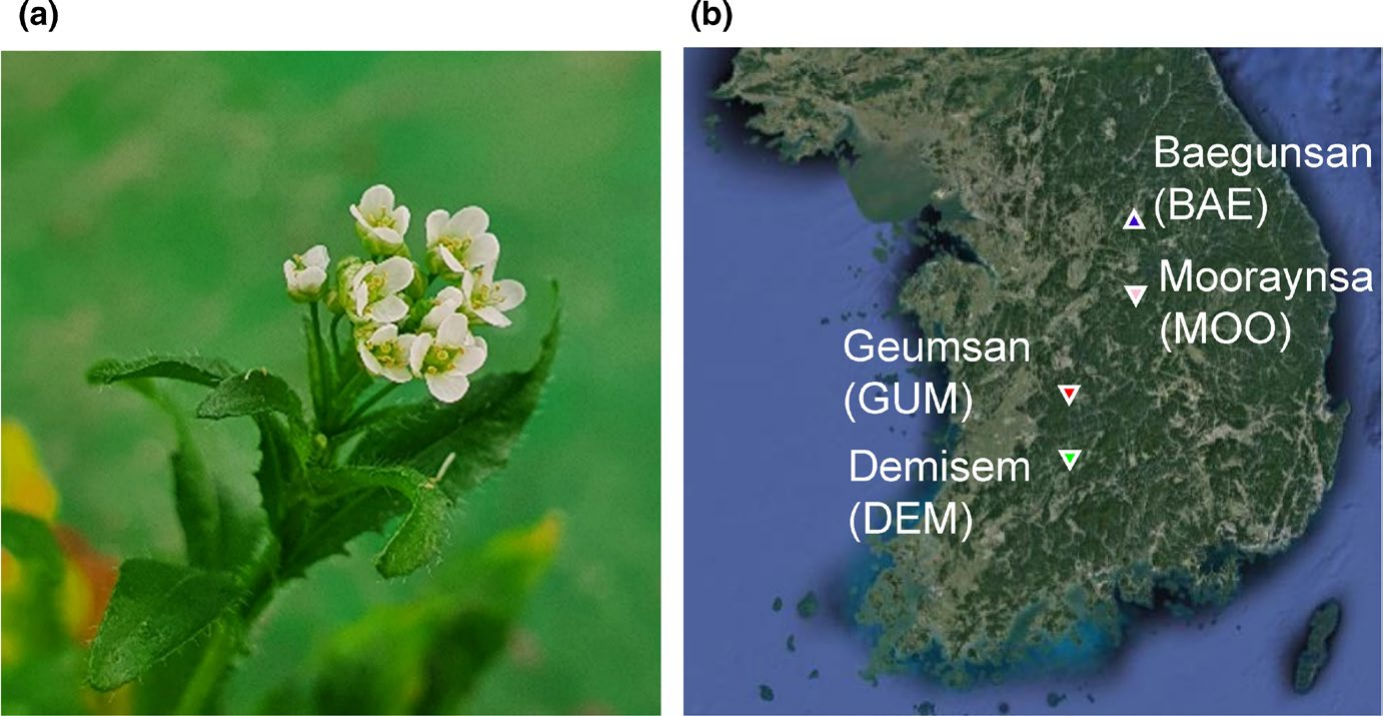
A photograph of *Capsella bursa*‐*pastoris* (a) and seed sources (b) in the Korean Peninsula. The GPS coordinates of source populations are given in Appendix S1

We randomly chose four *C*. *bursa*‐*pastoris* natural populations along a latitudinal gradient in South Korea in 2015 (Figure 1, Appendix S1). Seeds were collected from at least 20 maternal genotypes in each population. Germinants of field‐collected seeds were grown for one generation in a growth chamber, and seeds from these plants were used to examine SEB. In detail, field‐collected seeds were sown in pots (8 cm × 7.5 cm × 6 cm) containing commercial soil medium (Shinsung Mineral Co. LTD, Kyunggi‐do, Korea). The soil medium consisted of cocopeat (51.5% v/v), peat moss (10.0% v/v), zeolite (10.0% v/v), perlite (15.0% v/v), and vermiculite (13.0% v/v) with final pH of 5.0–7.0. Pots were maintained in a growth chamber at 25°C and a 12‐hour light/dark photoperiod with 100 μmol/s/m^2^ photosynthetically active radiation (PAR). Humidity inside the chamber was not controlled.

We randomly chose seven to eight maternal genotypes from each of the four *C*. *bursa*‐*pastoris* populations and sterilized seed surfaces following Johnston‐Monje and Raizada (2011) with modification. Briefly, seeds were soaked in 0.1% Triton X‐100 for 1 min, 3% sodium hydrochloride for 1 min, 70% ethanol for 1 min, and then washed three times with sterile distilled water. Surface sterilization was confirmed by incubating the sterilized seeds on potato dextrose agar (PDA) (# 213400, Difco Laboratories, Franklin Lakes, NJ, USA), R2A (# 218263, Difco), and Luria–Bertani (LB) agar (#7279, Acumedia, Lansing, MI, USA) at 25°C for a week. Only seeds that did not produce any microbial colonies were used to isolate bacterial endophytes and examine endophytic communities.

In order to visually confirm the occurrence of SEB, we used a fluorescence in situ hybridization (FISH) technique following Hewitson et al. (2010). Surface‐sterilized seeds were fixed with 4% paraformaldehyde. Seeds were embedded in the frozen section compound (FSC 22 Clear, Leica) at – 80℃ for 24 h, sectioned with 5 μm thickness using freezing microtome (DE/HM525NX, Thermo‐Fisher Scientific, Waltham, MA, USA), and attached on a clear slide glass. The whole slide glass was pre‐incubated in the hybridization buffer (0.9 M NaCl, 50 mM sodium phosphate (pH 7.0), 5 mM EDTA, 0.1% SDS, 0.5 mg of poly(A) per ml, 10× Denhardt solution, 35% (v/v) of formamide) at 45℃ for 30 min. After adding 50 ng of oligodeoxynucleotide probe (EUB338) with dye, the slide glass was incubated in the hybridization buffer for 2 h at 45℃. The slide was in the washing solution (0.9 M NaCl, 0.1% SDS, 20 mM Tris hydrochloride (pH 7.2)) for 30 min at 48℃ and washed twice with sterile distilled water. Pictures were taken using a Fluorescence microscope (EPI‐Fluorescence & DIC Microscope, Carl Zeiss, Oberkochen, Germany) and edited using the iSolation FL auto program.

### 2.2 Amplicon sequencing and clustering into operational taxonomic units (OTUs)

Equal amounts of seeds from seven individuals of each population were mixed to make three 500‐mg samples. Each seed sample was homogenized using TissueLyser II (QIAGEN, Hilden, Germany). DNA was extracted from each sample using DNeasy Plant Mini Kit (QIAGEN), and bacterial 16S rDNA was amplified. We used two methods to reduce chloroplast and mitochondrial DNA in the amplicon. First, we enriched bacterial cells in the homogenized seed mixture as described by Ikeda et al. (2009). Second, we amplified 16S rDNA using 799F (AACMGG‐ATTAGATACCCKG) and 1193R (ACGTCATCCCCACCTTCC). As shown by Beckers et al. (2016), the 799F – 1193R primer set amplifies mitochondrial and bacterial 16S rDNA but does not amplify chloroplast 16S rDNA. In addition, the size of amplified mitochondrial DNA (800 base pairs) was longer than that of bacterial DNA (approximately 450 bp). Amplified bacterial DNA was extracted from the gel and sequenced using the Illumina MiSeq platform by Macrogen Inc. (Seoul, Korea).

Sequence reads were processed with the Mothur v.1.43 pipeline following MiSeq SOP (Schloss et al., 2009). To trim sequence reads, we used *screen*.*seqs* with the options of zero maxambig, five maxhomop, and 430 maxlength. This procedure produced 86,980 high‐quality reads from 1,307,899 raw reads. Contigs were clustered into OTUs with 97% sequence identity and aligned using the SILVA *nr_132* database (Quast et al., 2012). Approximately 20% of the raw sequence reads were removed because they were chimeric or nonbacterial sequences including chloroplast, mitochondria, archaea, and eukaryotes. Singletons were removed by *remove*.*seqs* in Mothur (Allen et al., 2016). OTUs other than cyanobacteria were designated in the phyla or classes of Proteobacteria for further statistical analyses following Beckers et al. (2016).

### 2.3 Bacterial isolation and identification

**FIGURE 2:**
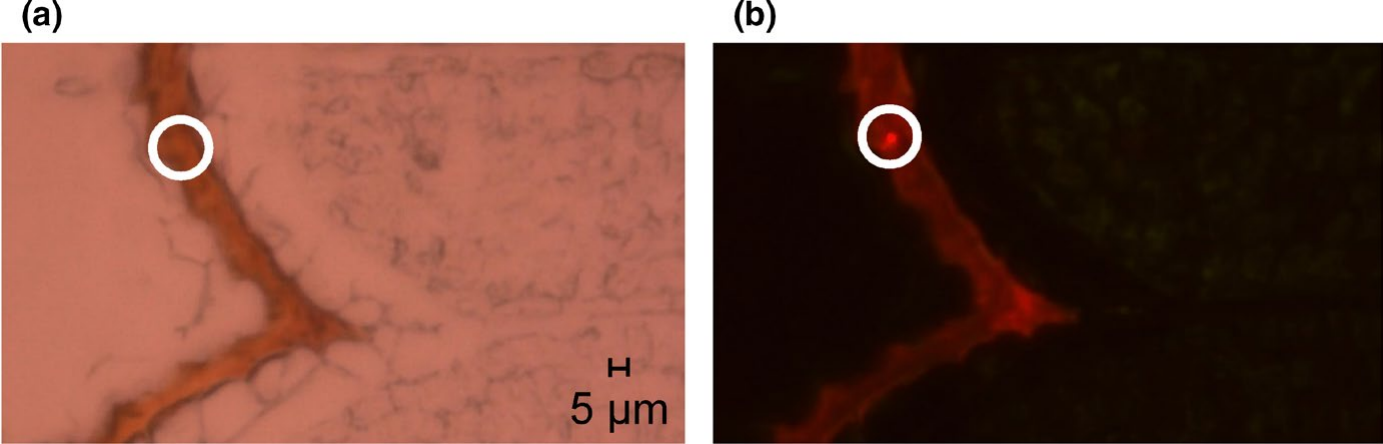
Images of fluorescent in situ hybridization (FISH) to detect seed endophytic bacteria inside seeds of DEM population. Seeds were sectioned using Cryomicrotome, and the EUB338 probes (Macrogen, Daejeon, Korea) were hybridized. The white circle in the right image indicates the position of endophytic bacteria. (a) is an image magnified 400^−1^ times by a light microscope. (b) is an image magnified 400× times with a fluorescence microscope

To isolate SEB (Figure 2), surface‐sterilized seeds were ground using a sterile mortar and pestle. The material was spread on five different solid media: LB agar, LGI (50 g/L sucrose, 0.01 g/L ferric chloride hexahydrate, 0.8 g/L potassium phosphate tribasic, 0.2 g/L magnesium sulfate heptahydrate, 0.002 g/L sodium molybdate dihydrate, 7.5 g/L agar; pH 7.5) for diazotrophic bacteria, R2A for oligotrophic bacteria, King's B agar (#60786, Sigma‐Aldrich, St. Louis, MO, USA), and commercial agar used for cooking for slow‐growing bacteria based on previous studies (Gagne‐Bourgue et al., 2013; Johnston‐Monje & Raizada, 2011). The plates were incubated at 25°C for a month during which a bacterial colony larger than 2 mm was picked for subculture (Gagne‐Bourgue et al., 2013). A total of 18 morphologically different colonies from the plates were selected, subcultured twice, and then preserved in 20% glycerol stock solutions at –80°C until required.

For bacterial DNA extraction, a single colony was inoculated into liquid ISP2 medium in a round‐bottom tube and incubated in a shaking incubator at 200 rpm at 25°C. DNA was extracted using Exgene Cell SV kits (Geneall, Seoul, Korea) following the manufacturer's instructions. The 16S rRNA gene was amplified using universal primers 27F (5′‐AGAGTTTGATCMTGGCTCAG‐3′) and 1492R (5′‐TACGGYTACCTTGTTACGACTT‐3′) (Coombs & Franco, 2003). Each PCR reaction contained a total of 50 μl consisting of 5 μl of 10× nTaq buffer, 5 μl of 5 μM dNTP, 1 U of nTaq polymerase (Enzynomics, Daejeon, Korea), 5 μl of each primer set, and 200 ng of template DNA. The reaction conditions were previously described (Coombs & Franco, 2003). PCR products were purified using the EZ‐pure PCR purification kit (Enzynomics) and sequenced (Macrogen Inc.). To identify bacterial isolates, we aligned nucleotide sequences using MEGA 6.0 (Tamura et al., 2013) and compared them with previously reported sequences of bacterial type strains using EZBiocloud (Chunlab, Seoul, Korea). A bacterial isolate was assigned to a species with the highest 16S rDNA sequence similarity. The 16S rDNA sequences of the isolates have been deposited in NCBI GenBank, and their accession numbers are given in Appendix S2.

### 2.4 Plant growth‐promoting traits

We assessed four microbial traits that were suggested to promote plant growth directly (Glick, 2012). For assays, isolated bacterial strains were individually grown in test tubes containing 7 ml LB medium (#7178, Acumedia) at 28°C in a shaking incubator (210 rpm) for three days. Because all PGP assay procedures include incubation of bacterial strains at 30°C (see below), cells were cultured at 28°C before the assay to acclimate cells to 30°C. The bacterial cells were harvested by centrifugation and rinsed with sterilized deionized water (DW) twice. The cells were suspended in DW to the optical density (OD) of 1.2 at 600 nm, and triplicates of cell suspension were used for all assays.

The ability to solubilize inorganic phosphate was examined following Nautiyal (1999). Briefly, 20 μl of prepared bacterial solution was inoculated in 8 ml of National Botanical Research Institute's Phosphate (NBRIP) growth medium (10 g/L glucose, 5 g/L MgCl_2_·6H_2_O, 0.25 g/L MgSO_4_·7H_2_O, 0.2 g/L KCl, 0.1 g/L (NH_4_)_2_SO_4_; pH 6.75) containing insoluble tricalcium phosphate (5 g/L Ca_3_(PO_4_)_2_), and incubated at 30°C in a shaking incubator (150 rpm) for two days. NBRIP without bacterial inoculation was prepared as a negative control. Clear supernatant (100 μl) after centrifugation was transferred to a new clean glass tube with 4.2 ml of sterile DW, 500 μl of 2.5% ammonium molybdate in 5 N sulfuric acid, and 200 μl of α‐amino‐naphthol solution. The mixture was incubated at room temperature for 30 min, and their absorbance at 660 nm was measured using BioSpectrometer basic (Eppendorf, Hamburg, Germany). The phosphate level was estimated based on a standard curve that ranged from 0.1 to 2 mM of phosphate.

Siderophore production was quantitated following Schwyn and Neilands (1987) and Murakami et al. (2021). Prepared bacteria solution (20 μl) was inoculated in 8 ml of the 10^−2^ diluted LB broth and incubated at 30°C in a shaking incubator (150 rpm) for four days. The supernatant (1 ml) was transferred to a glass tube with 700 μl of Chrome Azurol S (CAS) solution (0.165 g/L CAS, 0.082 g/L FeCl_3_, 0.397 g/L hexadecyltrimethylammonium bromide (HDTMA), in 100 mM piperazine buffer (pH 6.0) with 4 mM 5‐sulfosalicylic acid). After incubation for one hour at room temperature, the absorbance was measured at 630 nm using the Biospectrometer basic. The percent siderophore unit (psu) was calculated as (Ar/As)/Ar × 100 where Ar is the absorbance of CAS solution mixed with uninoculated media and As is the absorbance of CAS solution mixed with the supernatant of each sample culture.

We quantitated IAA following Patten and Glick (2002) with modification. We inoculated 20 μl of a bacterial solution to 8 ml of LB broth containing 1000 mg/L of L‐tryptophan (Sigma‐Aldrich) and incubated the broth at 30°C in a shaking incubator (150 rpm) for two days. LB broth without bacterial inoculation was prepared as a negative control. After centrifugation, 1 ml of supernatant was recovered and mixed with 4 ml of Salkowski's reagent (11.5 M H_2_SO_4_, 9.2 mM FeCl_3_, in sterilized DW). After incubation for 30 min at room temperature, the absorbance at 535 nm was measured. IAA concentration was estimated based on a standard curve that ranged from 1 to 50 μg/ml IAA (Duchefa Biochemie, Haarlem, The Netherlands).

ACC deaminase activity was determined following Penrose and Glick (2003) with modification. We inoculated 20 μl of bacterial solution into 7 ml of DF media (6 g/L Na_2_HPO_4_, 4 g/L KH_2_PO_4_, 2 g/L gluconic acid, 2 g/L citric acid, 0.2 g/L MgSO_4_•7H2O, 1 ml of trace element mixtures containing 0.001 g/L FeSO_4_•7H_2_O, 0.01 g/L H_3_BO_3_, 0.011 g/L MnSO_4_•H_2_O, 0.125 g/L ZnSO_4_•7H_2_O, 0.078 L^−1^ CuSO_4_•5H_2_O, 0.01 g/L MoO_3_) with 3 mM ACC (Sigma‐Aldrich) and glucose. After the incubation at 30°C in a shaking incubator (150 rpm) for four days, bacterial cells were collected by centrifugation, rinsed with saline solution three times, and Tris‐HCl (pH 7.5) one time. The pellet was suspended in 600 μl of 0.1 M of Tris‐HCl (pH 8.5) and mixed with 30 μl of toluene for 30 s. Each bacterial mixture (200 μl) was transferred to a new clean conical tube containing 20 μl of 0.5 M ACC and incubated at 30°C for 15 min. After 1 ml of 0.56 M HCl was added, the mixture was centrifugated to transfer 1ml of the supernatant to a new clean tube. A volume of 800 μl of 0.56 M HCl and 300 μl of 2, 4‐dinitrophenylhydrazine (0.2% 2,4‐dinitrophenylhydrazine in 2 M HCl) was added and incubated at 30°C for 30 min. After the addition of 2 ml of 2 N NaOH, the absorbance at 540 nm was measured using the BioSpectrometer basic. The concentration of α‐ketobutyrate was estimated based on a standard curve that ranged from 0 to 2 mM of α‐ketobutyrate (Sigma‐Aldrich). The quantity of the whole protein was estimated using Lowry's methods (Lowry et al., 1951).

### 2.5 *In vitro* growth assay

To confirm whether the isolated endophytic bacteria are able to promote plant growth, we grew plant seedlings and isolated bacteria together in a single plate. Seedlings germinated from surface‐sterilized seeds were used following previous studies (Dovana et al., 2015; Maggini et al., 2020). First, we examined the effects of bacteria on the model plant species, *Arabidopsis thaliana* Col line, as other studies often tested bacterial PGP activity using model plant species. After surface sterilization, seeds of *A*. *thaliana* were sown on a square plate with 0.2× Murashige and Skoog (MS) agar (#M022, Duchefa Biochemie, Haarlem, The Netherlands) containing 0.02% glycine, 0.5% inositol, and 0.5% sucrose. The plates were covered with aluminum foil and incubated at 25°C in the light for 16 h and 15°C in the dark for 8 h. Five days after incubation, 16 seedlings with a length of 1–2 cm were transferred to a new MS agar plate. The plates were incubated under the same conditions of the seed germination. Each bacterial isolate was streaked 7 cm below the root endpoint of seedlings in each plate, so that bacterial colonies did not contact plant seedlings (Figure 5e). Among 13 isolated bacteria, nine isolates were used for the experiment since four of them seldom grew in the MS agar. A plate that was not inoculated with any bacterial strain was a control. One‐third of a Petri dish was not covered with aluminum foil for plants to receive light. Shoot and root lengths of seedlings were measured using a digital caliper at the time of streaking bacteria in each plate and after five days of incubation. The growth was calculated as the shoot or root length measured five days after bacterial inoculation minus those measured before inoculation.

We conducted the same procedures using *C*. *bursa*‐*pastoris* seedlings. Seeds of ten maternal genotypes from each natural population were mixed and germinated in the same agar plates used for *A*. *thaliana*. Each plate had four seedlings from each plant population, resulting in a total of 16 seedlings. The plate inoculated with each bacterial isolate and a control plate without bacterial strain was replicated five times.

### 2.6 Statistical analyses

All statistical analyses were performed using R version 3.6.1 (R Core Team, 2019). The *vegan* package was used for the analysis of the bacterial community. The abundance of the phyla/classes was Hellinger transformed to reduce the weighting of highly abundant phyla/classes and the overweighting of rare phyla/classes. Unclassified sequence reads at the phylum level were removed from the dataset. The Bray–Curtis dissimilarity matrix was calculated based on the Hellinger transformed abundance data. To examine the variable composition of the seed bacterial community among the four populations, we conducted a permutational analyses of variance (PERMANOVA) with 9999 permutations and analyses of similarity (ANOSIM) with 9999 permutations. Nonmetric multidimensional scaling (NMDS) was conducted using the Bray–Curtis dissimilarity distances. To identify bacterial phylum/class contributing to the differentiation of seed endophytic communities, we compared the composition of each phylum/class among source populations using one‐way analyses of variance (ANOVA) with the population as a factor after arcsine transformation to satisfy normality. Pairwise comparisons between source populations were evaluated using Tukey's method in the *multcomp* package. As an alpha‐diversity measurement, the Inverse Simpson index was calculated based on the number and abundance of OTUs for each population. Tukey's multiple comparison was conducted to compare the diversity indexes among natural populations.

We conducted separate ANOVA for *A*. *thaliana* and *C*. *bursa*‐*pastoris* to evaluate the effect of bacterial inoculation on shoot and root growth. One‐way ANOVA was performed for *A*. *thaliana*, and the model included bacterial isolate as a fixed factor. For *C*. *bursa*‐*pastoris*, mixed model ANOVA was conducted with the source population, bacterial species, and their interaction as independent variables and the plate as a random factor. The effect of each isolated bacterium was evaluated by post hoc multiple comparisons between the plant growth with and without bacterial strain, and the statistical significance was adjusted based on Dunnett's method.

## 3 RESULTS

### 3.1 Endophytic bacterial communities of seeds in *C. bursa‐pastoris* natural populations

Sequencing of amplicon libraries generated a total of 1,307,889 reads with a mean of 87,013 sequences per sample. Approximately 20% of the total sequences were of chloroplast or mitochondrial DNA, and approximately 80% of the remaining sequences were cyanobacteria or unclassified bacteria. The bacterial reads were assigned to 82 OTUs at a 97% cut‐off in the Mothur pipeline. Rarefaction curves suggested that the dataset might be inadequate to capture the bacterial communities fully (Appendix S3), but the Goods’ coverage estimates of all samples were over 99% (Appendix S3).

**FIGURE 3:**
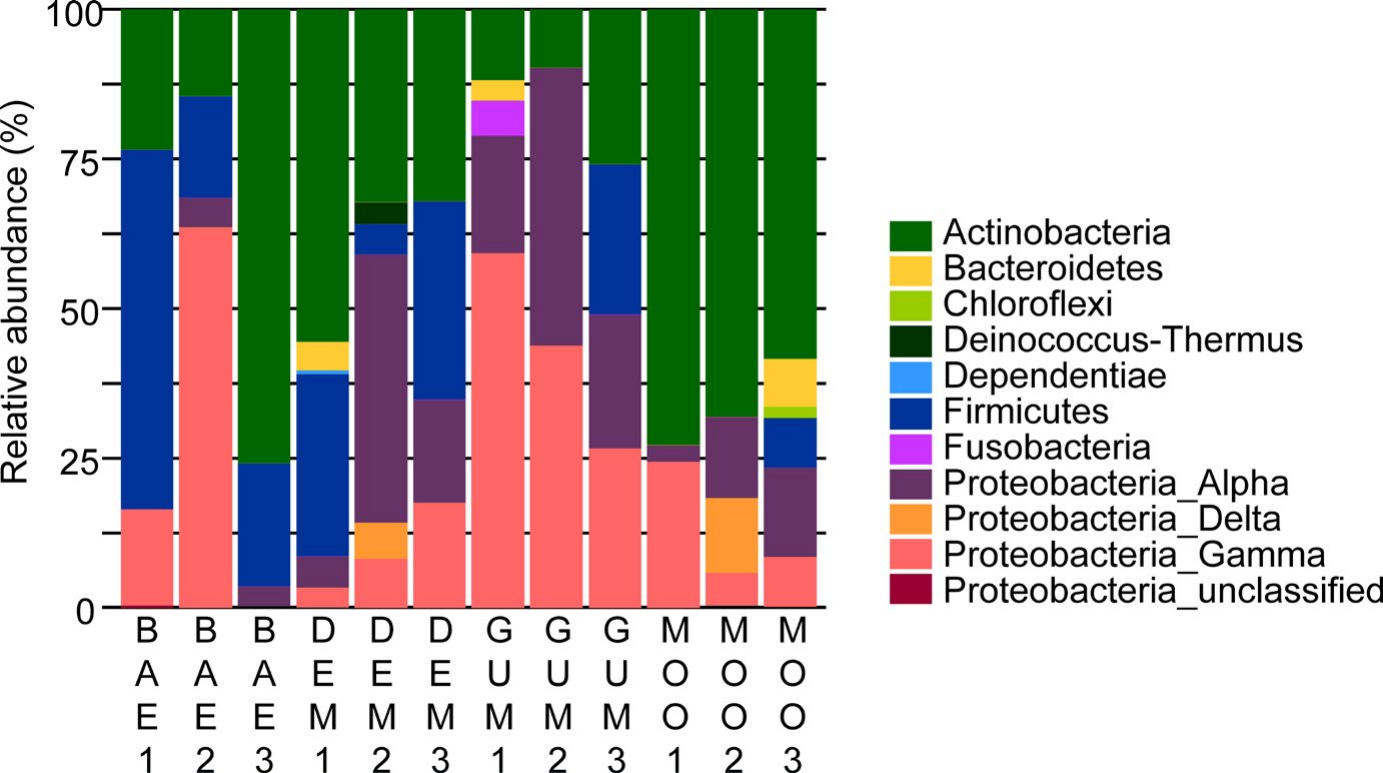
Relative sequence abundance of bacterial phyla from seeds of four *Capsella bursa*‐*pastoris* natural populations. Proteobacteria are shown at the class level following Beckers et al. (2016), and replicates are presented separately. Abbreviations of plant populations are given in Figure 1

OTUs were assigned to eight phyla: Actinobacteria, Bacteroidetes, Chloroflexi, Deinococcus, Dependentiae, Firmicutes, Fusobacteria, and Proteobacteria. Although not evenly distributed, Actinobacteria¸ Firmicutes, Alphaproteobacteria, and Gammaproteobacteria occurred most commonly across four plant populations (Figure 3). Results of PERMANOVA (*F* = 2.67, *p* < .05) and ANOSIM (*R* = 0.35, *p* < .05) indicated that *C*. *bursa*‐*pastoris* natural populations had a differential composition of seed endophytic phyla/classes. In particular, NMDS plot indicated that the bacterial community was likely divided into three groups at the phylum/class level, population BAE, population GUM, and population DEM and MOO. This was supported by the results of ANOSIM (*R* = 0.34, *p* < .05).

Additional ANOVA for each phylum showed that the composition of Actinobacteria (*F* = 3.72, *p* = .06) and Alphaproteobacteria (*F* = 3.48, *p* = .07) differed among plant populations (Appendix S4). In particular, the population GUM had a lower proportion of Actinobacteria (*t* = 3.34, *p* = .04) than the population MOO, and a higher proportion of Alphaproteobacteria (*t* = 3.02, *p* = .06) than the population BAE.

**FIGURE 4:**
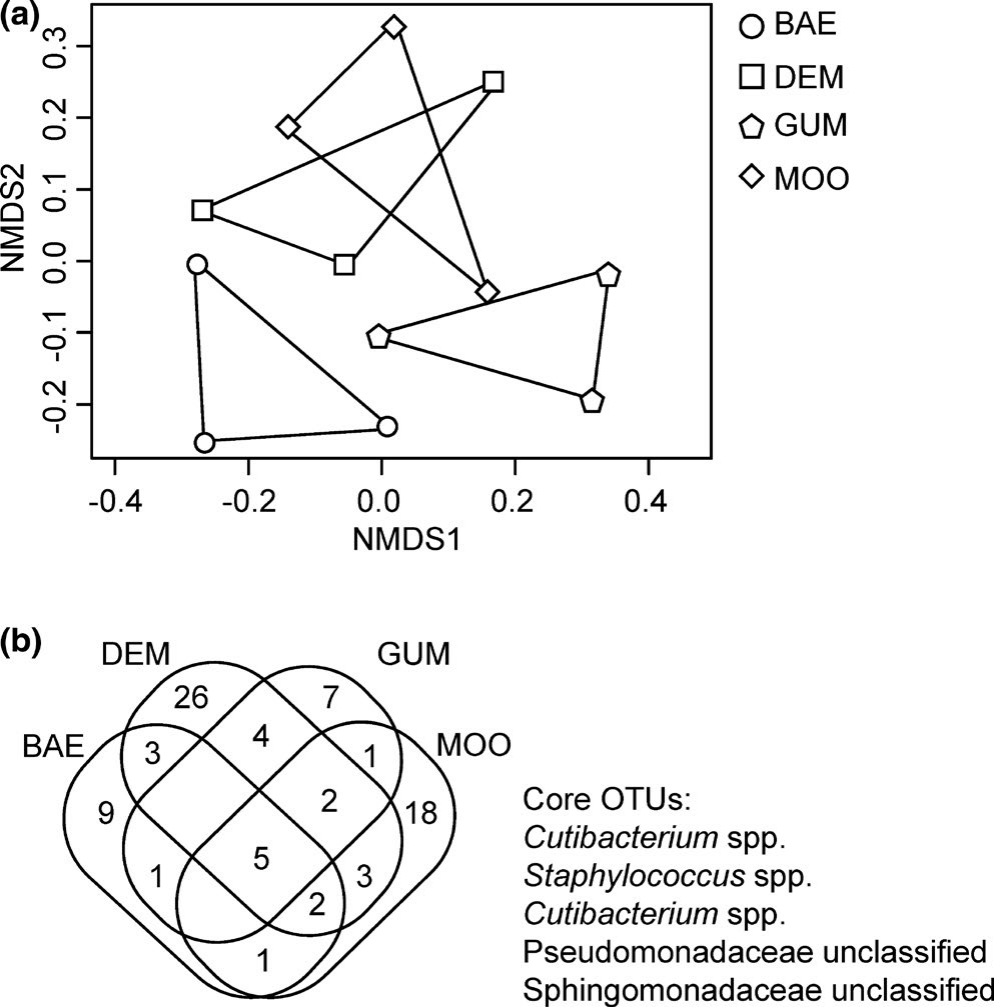
Differential bacterial communities among *Capsella bursa*‐*pastoris* natural populations. (a) is a nonmetric multidimensional scaling plots of endophytic bacterial communities in four *C*. *bursa*‐*pastoris* natural populations. The Bray–Curtis dissimilarity distances were calculated from the relative abundance of phyla and classes of Proteobacteria and used for cluster analysis. (b) is a Venn diagram showing the number of OTUs that occurred in single or multiple populations

The number of OTUs from each sample varied from 21 to 45 (Figure 4b, Appendix S4). The Inverse Simpson index differed among endophytic communities of plant populations (F = 4.58, *p* < .05). Tukey's multiple comparisons showed that the endophytic community of the population GUM exhibited a higher Inverse Simpson index than that of population BAE (*t* = 3.015, *p* = .07) and population MOO (*t* = 3.072, *p* = .06). Although the population MOO had the largest number of OTUs, its inverse Simpson index was the lowest (Appendix S4). Sixty of 82 OTUs occurred only in a single plant population (Figure 4b), suggesting that both the species composition as well as the proportion of seed endophytic communities differed among natural plant populations.

### 3.2 Isolated endophytic bacteria and their PGP traits

A total of 13 cultivable bacterial species in six genera were isolated from four *C*. *bursa*‐*pastoris* natural populations based on the similarities of partial 16S rDNA sequences (1203 – 1439 nucleotides) (Table 1). All isolates showed a high homology of 97% to 100% with previously known sequences. *Streptomyces* and *Bacillus* were the most dominant genera in the culture‐dependent method. At the species level, four of five *Streptomyces* species and one of two *Bacillus* species occurred only in a single plant population.

**TABLE 1:** Endophytic bacteria isolated from natural populations of *C*. *bursa*‐*pastoris* and results of plant growth‐promoting (PGP) assays

Source population	Strains	The closest type strain (accession number)	Similarity (%)	Plant growth‐promoting activity			
				ACC deaminase activity (nmol α‐KB/mg protein/h)	IAA production (µg/ml)	Siderophore production (psu, % of siderophore units)	Phosphate solubilization (mg/L of PO_4_)
BAE	B1	*Bacillus aryabhattai* B8W2w (EF114313)	1366/1374 (99.42)	30.86 ± 3.59	26.37 ± 0.34	20.66 ± 0.46	624.31 ± 26.91
	B2	*Rhodococcus corynebacterioides* DSM 20151 (AF430066)	1322/1329 (99.47)	22.09 ± 1.18	–	3.57 ± 0.19	22.39 ± 1.03
DEM	D1	*Streptomyces qinglanensis* 172205 (HQ660227)	1338/1339 (99.93)	2.09 ± 0.29	22.96 ± 0.68	26.06 ± 0.16	465.08 ± 60.35
	D2	*Streptomyces olivaceus* NRRL B‐3009 (JOFH01000101)	1337/1338 (99.92)	19.49 ± 1.08	22.17 ± 0.74	27.93 ± 0.10	499.77 ± 18.74
	D3	*Streptomyces griseoplanus* NRRL B‐3064 (AB184138)	1307/1322 (98.87)	1.88 ± 0.31	–	4.15 ± 0.29	18.92 ± 2.71
	D4	*Staphylococcus haemolyticus* MTCC 29970 (LILF01000056)	1363/1363 (100)	20.10 ± 0.26	4.36 ± 0.42	1.87 ± 0.33	12.30 ± 0.89
	D5	*Paenibacillus tritici* RTAE36 (CP009285)	1373/1373 (99.12)	21.63 ± 0.53	–	1.52 ± 0.37	22.39 ± 1.36
	D6	*Bacillus altitudinis 41KF2b (*ASJC01000029)	1288/1289 (99.92)	2.00 ± 0.16	–	1.56 ± 0.16	14.50 ± 2.46
GUM	G1	*Streptomyces qinglanensis* strain 172205 (HQ660227)	1332/1333 (99.92)	1.40 ± 0.20	20.02 ± 1.27	28.92 ± 0.62	524.05 ± 37.66
	G2	*Rhodococcus cercidiphylli* YIM 65003 (EU325542)	1203/1220 (98.61)	24.07 ± 0.77	–	28.34 ± 0.31	501.03 ± 23.63
	G3	*Streptomyces anulatus* NRRL B‐2000 (DQ026637)	1329/1329 (100.00)	0.94 ± 0.09	21.65 ± 0.46	23.11 ± 1.00	388.15 ± 26.31
	G4	*Bacillus altitudinis* 41KF2b (ASJC01000029)	1353/1354 (99.93)	1.16 ± 0.05	–	3.97 ± 0.35	8.83 ± 3.63
	G5	*Paenibacillus agarexedens* DSM 1327 (KF479658)	1377/1439 (97.55)	1.24 ± 0.03	–	2.72 ± 0.36	11.67 ± 1.43
MOO	M1	*Streptomyces albus* NBRC 13014 (NR118467)	1242/1242 (100.00)	0.88 ± 0.05	–	28.11 ± 0.35	278.43 ± 14.05
	M2	*Pseudomonas geniculata* ATCC 19374 (AB021404)	1307/1309 (99.85)	1.13 ± 0.09	34.32 ± 0.80	3.03 ± 0.48	31.22 ± 9.86

Note

Type strains with the highest 16S rDNA sequence similarity (GenBank accession number) are given. –, not detected.

Eleven out of fifteen isolates expressed variable PGP characteristics (Table 1). Compared to other bacterial strains, *Bacillus aryabhattai* B1 and *Streptomyces* (*St*.) *olivaceus* D2 produced a relatively high amount of PGP molecules in all testing assays. In contrast, *Streptomyces griseoplanus* D2, *Bacillus altitudinis* D6, *B*. *altitudinis* G4, and *Paenibacillus agarexedens* G5 exhibited low activities in all assays. *Streptomyces* strains except *St*. *griseoplanus* D3 produced more than 20 µg/ml IAA, 23% of siderophore units, and 278 mg/L soluble phosphate. *Rhodococcus corynebacterioides* B2, *Staphylococcus haemolyticus* D4, *Paenibacillus tritici* D5 exhibited ACC deaminase activity higher than 20 nmol α‐KB/mg protein/h, but they showed low activity in other assays. Endophytic communities from three plant populations had at least one bacterial strain that exhibited positive activity in four PGP trait assays.

### 3.3 Growth responses of seedlings to endophytic bacteria

**FIGURE 5:**
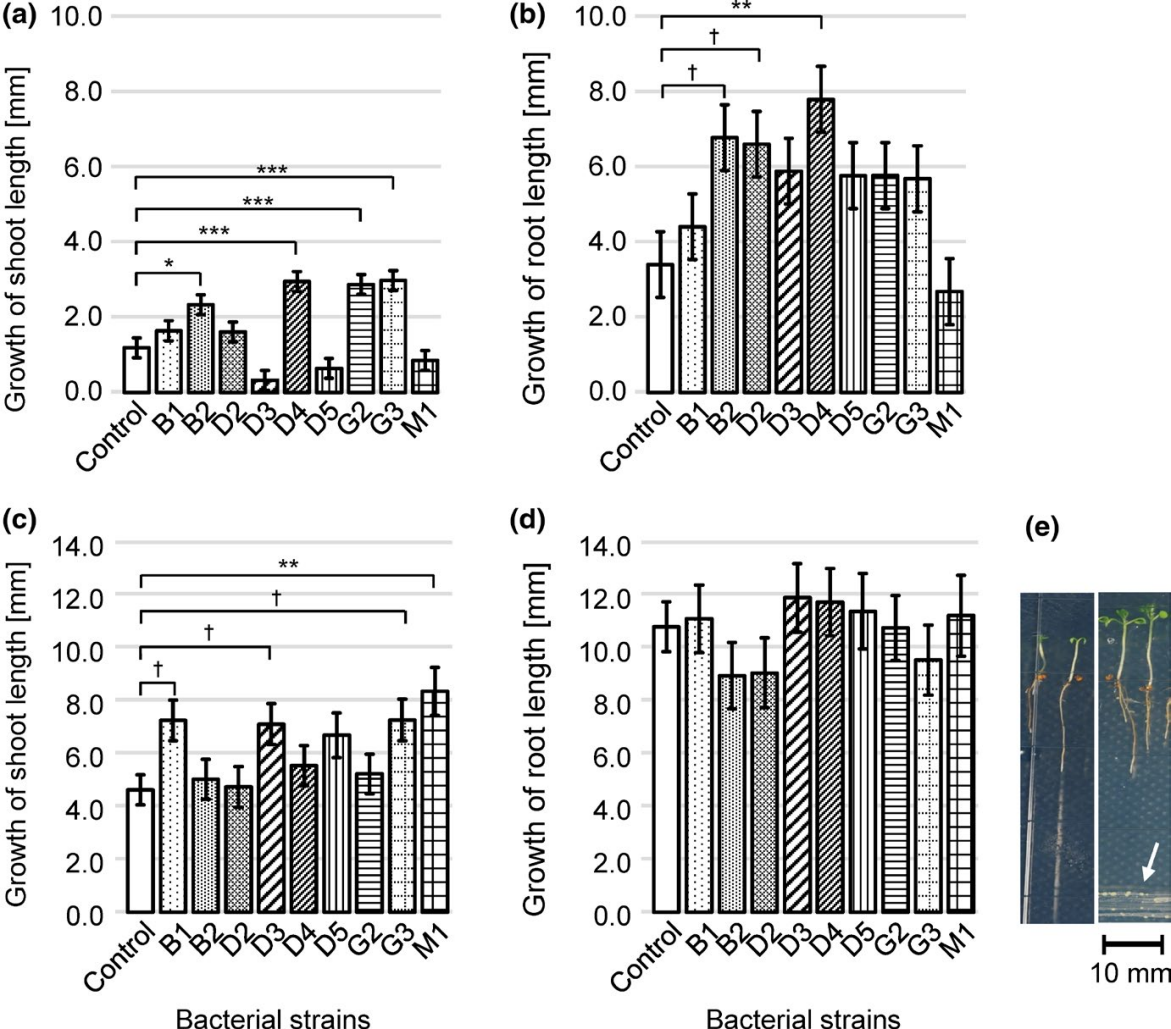
Responses of plant seedlings to isolated bacterial endophytes in the *in vitro* plate assays. The shoot and root growth was calculated as the difference in shoot and root length during a 5‐day incubation of seedlings and a bacterial strain in the same plate. Averages of shoot and root growth and standard errors are given. (a) and (b) are the responses of *Arabidopsis thaliana* and (c) and (d) are the responses of *Capsella bursa*‐*pastoris*. (e) is photographs of *C*. *bursa*‐*pastoris* grown with (right) and without (left) *B*. *aryabhattai* inoculation (arrow). Abbreviations of bacterial strains are given in Table 1. Dunnett's multiple comparison was conducted between the control and each inoculated bacterium, and the significance levels of the differences are given. †*p* < .10; *p < .05, **p < .01, ***p < .001

Isolated bacterial strains affected shoot (*F* = 14.07, *p* < .001) and root growth (*F* = 3.18, *p* < .01) of *A*. *thaliana* (Figure 5a,b). *A*. *thaliana* grown with *Rhodococcus corynebacterioides* B2 (*Z* = 3.04, *p* < .05), *Staphylococcus haemolyticus* D4 (*Z* = 4.68, *p* < .001), *Rhodococcus cercidiphylli* G2 (*Z* = 4.47, *p* < .001), and *Streptomyces anulatus* G3 (*Z* = 4.76, *p* < .001) exhibited higher shoot growth than plants without bacteria (Figure 5a). Among the four isolates, *R*. *corynebacterioides* B2 (*Z* = 2.72, *p* = .05) and *S*. *haemolyticus* D4 (*Z* = 3.54, *p* < .01) stimulated root growth (Figure 5b).

Similar to *A*. *thaliana*, shoot growth of *C*. *bursa*‐*pastoris* was affected by isolated seed endophytes (*F* = 2.91, *p* < .01), but the root growth was not (*F* = 0.69, *p* = .71) (Figure 5c,d). The plant population by isolate interaction was not statistically significant in either shoot (*F* = 0.6, *p* = .94) and root growth (*F* = 1.14, *p *= .29), indicating that the effect of isolates on the shoot and root growth was similar across the tested plant populations. When averaged across natural populations, at least one strain from each population stimulated shoot growth of *C*. *bursa*‐*pastoris* (*Bacillus aryabhattai* B1, *Z* = 2.74, *p* = .06; *St*. *griseoplanus* D3, *Z* = 2.58, *p* = .08; *St*. *anulatus* G3, *Z* = 2.72, *p* = .06; *St*. *albus* M1, *Z* = 3.45, *p* < .01) (Figure 5c). One of them, *St*. *anulatus* G3 also increased the growth of *A*. *thaliana* (Figure 5).

## 4 DISCUSSION

### 4.1 Differences in seed endophytic communities among natural populations

The major phyla inside the seeds of *C*. *bursa*‐*pastoris* were Actinobacteria, Firmicutes, and Proteobacteria, which are also major bacterial phyla in other plant species (Card et al., 2015; Truyens et al., 2015). The number of OTUs and sequence reads were comparable to the values in previous studies examining seed bacterial communities in other plant species (Liu et al., 2019; Truyens et al., 2016).

Although plant species tend to have variable seed endophytic communities (Ikeda et al., 2009; Johnston‐Monje & Raizada, 2011), it has been inconclusive whether natural populations of the same species also harbor differential seed endophytic communities. In *C*. *bursa*‐*pastoris*, similar phyla constituted the seed bacterial community, but their proportion differed among natural populations (Figure 3). Sixty of 82 OTUs from the sequence analyses occurred only in a single population (Figure 4b), and so did 11 of 13 cultivable seed bacteria (Table 1). These results suggest that bacterial species composition and their proportion in the seed endophytic community differ among plant natural populations.

Diverse factors affect the seed endophytic community. Plant genotype has been proposed to potentially shape the seed bacterial community (Gagne‐Bourgue et al., 2013; Granér et al., 2003; Xu et al., 2014), while some studies could not detect its effect (Fürnkranz et al., 2012; Kukkurainen et al., 2005). In this study, the proportion of Actinobacteria differed between populations MOO and GUM (Figure 3). Notably, different morphological and physiological traits were observed between populations MOO and GUM when plants were grown in a common environmental condition, suggesting that the two populations likely consisted of different genotypes (Choi et al., 2019). Genotypes of *C*. *bursa*‐*pastoris* seem to affect the endophytic community of seeds.

Habitat difference in natural populations is another factor that potentially alters the seed endophytic community. For instance, environmental conditions such as soil type or environmental stress can change seed bacterial communities (Hardoim et al., 2012; Truyens et al., 2013). Soil microbial communities in plant habitats can additionally influence the seed endophytic community since bacteria in the soil can enter the plant body and migrate into the seeds (Bertani et al., 2016; Cope‐Selby et al., 2017). In this study, we grew *C*. *bursa*‐*pastoris* populations in the same growth chamber for one generation and used their seeds to examine endophytic bacterial communities. Thus, environmental factors would have a limited effect on the variable seed endophytic communities. In contrast, we cannot exclude the possibility that the natural habitats of our testing plant population might have differential soil bacterial communities, which might cause distinctive seed endophytic communities.

### 4.2 Isolated endophytic bacteria from *C. bursa‐pastoris* seeds and their PGP activities

Similar to previous studies, isolated cultivable bacterial strains partially represent OTUs observed by NGS analysis (Johnston‐Monje & Raizada, 2011). Most isolates were Actinobacteria and Firmicutes, which is consistent with the result of NGS analysis (Figure 3). In contrast, only one Gammaproteobacteria species, *Pseudomonas geniculate* M2, was isolated in the culture‐dependent method while Gammaproteobacteria was one of the dominant phyla in the NGS analysis.

Despite this limitation, only one (*S*. *haemolyticus* D4) out of fifteen isolates was assigned to bacterial species that have been previously reported as seed endophytes (Najnin et al., 2014). The other strains except *P*. *agarexedens* G5 were known to inhabit the rhizosphere or plant tissues other than seeds (Beneduzi et al., 2008; Gopalakrishnan et al., 2015; Hong et al., 2015; Hu et al., 2012; Kim et al., 2012; Qin et al., 2017; Verma et al., 2016; Wang et al., 2018). Given that SEB would be a subset of the bacterial community of phyllosphere or rhizosphere (Bertani et al., 2016; Cope‐Selby et al., 2017), our result suggests testing more plant species would expand the current database of SEB.

Eleven out of fifteen isolates exhibited positive activity in testing assays. In particular, half of isolates (*B*. *aryabhattai* B1, *St*. *qinglanensis* D1 and G1, *St*. *olivaceus* D2, *R*. *cercidiphylli* G2, and *St*. *albus* M1) exhibited the phosphate‐solubilizing activity that is comparable to other phosphate‐solubilizing bacteria (Khan et al., 2014). This study examined whether isolated strains could dissolve tricalcium phosphate to determine phosphate‐solubilizing activity. A limitation of this method is that it cannot measure the ability to dissolve metal phosphate, another common form of insoluble phosphate in soil (Bashan et al., 2013). Despite this limitation, the tricalcium phosphate method has been widely adopted in many studies as a useful screening method to identify candidate bacterial strains facilitating plant use of insoluble phosphates (Mahdi et al., 2021; Varga et al., 2020).

All bacterial isolates with phosphate‐solubilizing activity produced 20.66–28.92% unit siderophores. *Pseudomonas* strains producing a similar quantity of siderophore were shown to promote the shoot and root growth in rice, wheat, or bottle gourd (Agrawal et al., 2017). Siderophores excreted from bacterial cells can sequester ferric ions to facilitate plant absorption (Glick, 2012). In addition, siderophores can act as chelating agents for Ca^2+^ or metal ions that make phosphate ions insoluble (Wang et al., 2018). Siderophore production of isolates could synergistically contribute to their capacity of phosphate solubilization.

Six isolates exhibited a positive activity in the ACC deaminase assay. However, the value of ACC deaminase activity was lower than that of other *Streptomyces* spp. with 57–354 nmol α‐KB/mg protein/h or *Pseudomonas* spp. with 1400–40870 nmol α‐KB/mg protein/h (Agrawal et al., 2017; El‐Tarabily, 2008). In response to environmental stresses, the ethylene level in a plant increases to reduce plant growth (Pierik et al., 2006). ACC deaminase can block the ethylene production of plants by cleaving ACC, the immediate precursor of ethylene, into α‐ketobutyrate and ammonia (Glick, 2012; Penrose & Glick, 2003). Consequently, ACC deaminase can ameliorate an adverse effect of environmental stress on plant growth. Notably, winter annuals like *C*. *bursa*‐*pastoris* often exhibit the stress avoidance strategy, such that they finish their life cycle before facing stressful environmental conditions like hot and dry summer (Crawley, 1997). Thus, the advantage of winter annual plants might be small by harboring bacteria with high ACC deaminase activity. In an evolutionary ecological perspective, it would be of interest to examine whether plant life histories correlate with the abundance of SEB with ACC deaminase activities.

### 4.3 Bacterial strains stimulating the growth of *C. bursa‐pastoris* or *A. thaliana*

Four isolated strains (*B*. *aryabhattai* B1, *St*. *griseoplanus* D3, *St*. *anulatus* G3, and *St*. *albus* M1) stimulated shoot growth of *C*. *bursa*‐*pastoris* (Figure 5). In previous studies, strains of those species were isolated from the rhizosphere of plants and shown to promote the growth of crop plant species (Bhattacharyya et al., 2017; Boubekri et al., 2021; Subramaniam et al., 2020; Wang et al., 2018). This study showed that some strains of four species could migrate into seed.

It is well acknowledged that soil microbiome can facilitate weed establishment (Trognitz et al., 2016). However, the ecological significance of SEB in weedy species has gained attention just recently. For instance, SEB can contribute to the seed germination and growth in invasive *Phragmites australis* (White et al., 2018), drought resistance in *Lactuca serriola* (Jeong et al., 2021), and the competitive ability by antagonistic effects on the competitor species (Elmore et al., 2019). Although soil microbiome has been postulated as a major source of mutualistic microorganisms for weedy plant species (Trognitz et al., 2016), our results imply that SEB might be another source of mutualistic microorganisms.

Among four strains stimulating *C*. *bursa*‐*pastoris* shoot growth, *B*. *aryabhattai* G1, *St*. *anulatus* G3, and *St*. *albus* M1 produced siderophore and soluble phosphate (Figure 4, Table 1), suggesting those PGP traits likely play an important role in promoting the seedling growth. All those species are known to have the phosphate‐solubilizing ability (Bhattacharyya et al., 2017; Boubekri et al., 2021; Subramaniam et al., 2020). In contrast, one isolate (*St*. *griseoplanus* D3) exhibited low activities in all testing PGP substances. Given that the phosphate‐solubilization activity of *St*. *griseoplanus* depends on growth media (Wang et al., 2018), *St*. *griseoplanus* D3 might increase its activity in the Murashige and Skoog (MS) agar used for plant growth test. Alternately, other PGP mechanisms might contribute to plant growth. For instance, the production of gibberellins or volatile compounds is also suggested to enhance plant growth (Glick, 2012), although we did not test those characteristics.

Even though they are phylogenetically close relatives (Beilstein et al., 2008), seedlings of *A*. *thaliana* and *C*. *bursa*‐*pastoris* reacted differently to the same isolated strains. Given the complex interaction between host plant species and endophytic bacteria (Carvalho et al., 2016), the PGP activity of endophytic bacteria has been hypothesized to depend on the host plant species (Long et al., 2008). Interestingly, a few bacterial strains isolated from *C*. *bursa*‐*pastoris* exhibited the ability to promote the seedling growth of *A*. *thaliana*, suggesting that some endophytic bacteria can promote the growth of nonhost plant species. Endophyte host specificity does not necessarily exclude the possibility that endophytic bacteria can stimulate the growth of nonhost plant species. Although some endophytic bacteria could have PGP activities on a broad range of plant species, the mechanism of PGP activity likely differs among plant species (Long et al., 2008; Ma et al., 2011).

This study conducted in vitro seedling tests and showed that seven out of fifteen bacterial isolates stimulated shoot growth of either *C*. *bursa*‐*pastoris* or *A*. *thaliana*. However, it should be noted that in vitro tests might have a limited power to evaluate the diverse effects of endophytic bacteria on plant growth. For instance, we did not directly infect bacteria into individual plants, but the effects of endophytic bacteria might depend on their location inside the plant body and the developmental stage of the host plants (Truyens et al., 2015). In addition, the in vitro seedling test cannot evaluate plant tolerance to biotic and abiotic environmental stresses. We are conducting additional in vivo studies to examine plant performance in natural environments using seeds directly inoculated by isolated bacteria. The results are expected to provide more clear evidence on the interaction between endophytic bacteria and plant performance.

## 5 CONCLUSIONS

This study revealed that the bacterial communities inside seeds differed among *C*. *bursa*‐*pastoris* natural populations, indicating the diversity of SEB should be evaluated at the population level. Isolated bacteria from seeds produced a relatively high amount of PGP substances, and four strains stimulated shoot growth of *C*. *bursa*‐*pastoris*. This result implies that SEB in addition to soil microorganisms can be a source of mutualistic microorganisms facilitating seedling establishment that is a critical fitness component of weedy plant species.

## CONFLICT OF INTEREST

The authors declare no conflict of interest.

## AUTHOR CONTRIBUTION

**Byungwook Choi:** Conceptualization (equal); Data curation (lead); Formal analysis (lead); Methodology (lead); Writing – original draft (lead). **Seorin Jeong:** Data curation (supporting); Formal analysis (supporting); Methodology (supporting). **Eunsuk Kim:** Conceptualization (equal); Data curation (supporting); Funding acquisition (lead); Methodology (supporting); Supervision (lead); Writing – original draft (supporting); Writing – review & editing (lead).

## Supporting information

Appendix S1

Appendix S2

Appendix S3

Appendix S4

## Data Availability

All data and R code for the analyses for this article are available in Dryad (https://datadryad.org/stash/share/ANRIA2‐7vado2aIuuzvLhwwnrZVlNtdqcUCjPF5sLQ0, https://datadryad.org/stash/share/ANRIA2‐7vado2aIuuzvLhwwnrZVlNtdqcUCjPF5sLQ0).
