# Supplementary material for: Variation of the seed endophytic bacteria among plant populations and their plant growth‐promoting activities in a wild mustard plant species, Capsella bursa‐pastoris
Source: Ecol Evol. 2022 Mar 7;12(3):e8683. doi: 10.1002/ece3.8683 (PMC8901890; doi:10.1002/ece3.8683)
Supplement: Supplementary file 1 — Appendix S1 [file ECE3-12-e8683-s004.docx]

Appendix S1. Source populations of *C. bursa-pastoris* with their locations.

| Population | Location |
| --- | --- |
| Baegunsan (BAE) | N 37° 16´ 2.870´´, E 127° 57´ 19.050´´ |
| Moorayngsa (MOO) | N 36° 50´ 2.110´´, E 128° 1´ 33.439´´ |
| Geumsan (GUM) | N 36° 4´ 8.524´´, E 127° 22´ 6.269´´ |
| Demisem (DEM) | N 35° 39´ 12.228´´, E 127° 27´ 26.356´´ |
