# Supplementary material for: Variation of the seed endophytic bacteria among plant populations and their plant growth‐promoting activities in a wild mustard plant species, Capsella bursa‐pastoris
Source: Ecol Evol. 2022 Mar 7;12(3):e8683. doi: 10.1002/ece3.8683 (PMC8901890; doi:10.1002/ece3.8683)
Supplement: Supplementary file 2 — Appendix S2 [file ECE3-12-e8683-s003.docx]

Appendix S2. The accession number of partial 16S rDNA sequences of isolated bacteria from *C. bursa-pastoris* seeds.

| Source population | Strains | Accession number |
| --- | --- | --- |
| Baegunsan (BAE) | B1 | MT543134 |
|  | B2 | MT543221 |
| Demisem (DEM) | D1 | MT568572 |
|  | D2 | MT543222 |
|  | D3 | MT543225 |
|  | D4 | MT569983 |
|  | D5 | MT569978 |
|  | D6 | MT569984 |
| Geumsan (GUM) | G1 | MH517409 |
|  | G2 | MT569980 |
|  | G3 | MT569979 |
|  | G4 | MH517411 |
|  | G5 | MT568608 |
| Mooryangsa (MOO) | M1 | MT569982 |
|  | M2 | MT569981 |
