# Supplementary material for: Variation of the seed endophytic bacteria among plant populations and their plant growth‐promoting activities in a wild mustard plant species, Capsella bursa‐pastoris
Source: Ecol Evol. 2022 Mar 7;12(3):e8683. doi: 10.1002/ece3.8683 (PMC8901890; doi:10.1002/ece3.8683)
Supplement: Supplementary file 3 — Appendix S3 [file ECE3-12-e8683-s002.docx]

Appendix S3. Rarefaction curves and Goods’ coverage estimates of amplicons.

Figure S3.1. Rarefaction curves of seed endophytic communities from four *C. bursa-pastoris* populations. Three replicates of each population are shown separately. Blue, population BAE; green, population DEM; red, population GUM; pink, population MOO.


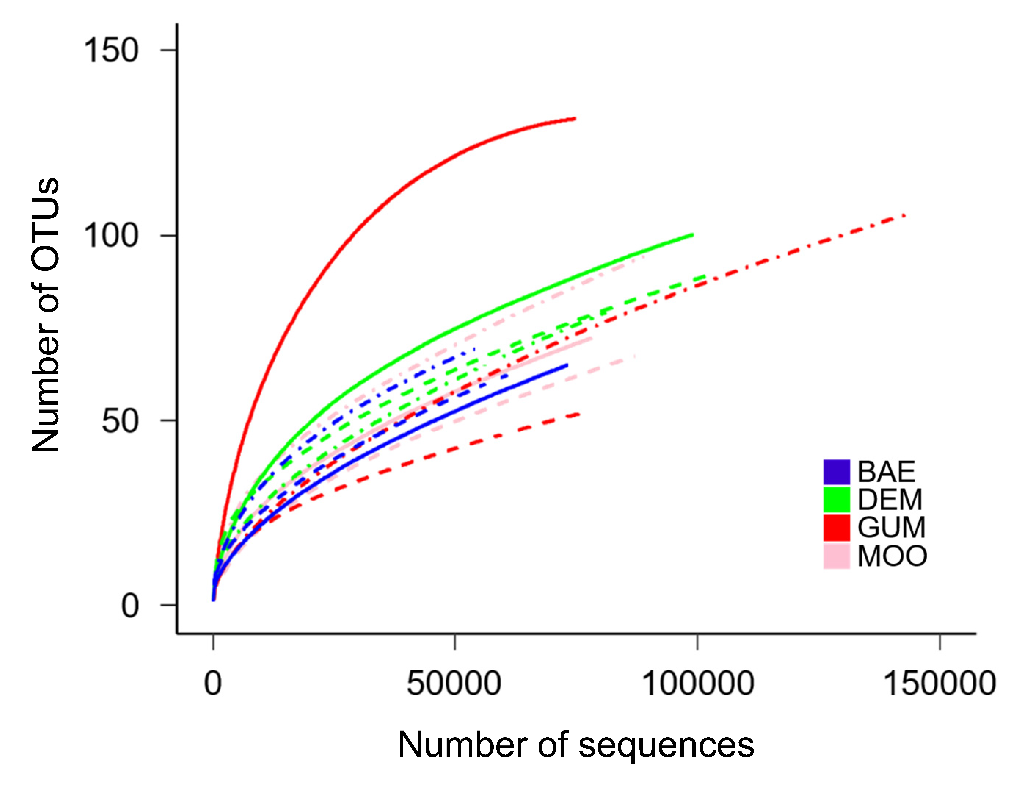


Table S3.2. Goods’ coverage estimates of three amplicon replicates from each *C. bursa-pastoris* population

| Source population | Sample | Good’s estimates (%) |
| --- | --- | --- |
| Baegunsan (BAE) | BAE1 | 99.9496 |
|  | BAE 2 | 99.9459 |
|  | BAE 3 | 99.9446 |
| Demisem (DEM) | DEM 1 | 99.9632 |
|  | DEM 2 | 99.9582 |
|  | DEM 3 | 99.9470 |
| Geumsan (GUM) | GUM 1 | 99.9839 |
|  | GUM 2 | 99.9662 |
|  | GUM 3 | 99.9593 |
| Mooryangsa (MOO) | MOO 1 | 99.9525 |
|  | MOO 2 | 99.9560 |
|  | MOO 3 | 99.9433 |
