# Supplementary material for: Variation of the seed endophytic bacteria among plant populations and their plant growth‐promoting activities in a wild mustard plant species, Capsella bursa‐pastoris
Source: Ecol Evol. 2022 Mar 7;12(3):e8683. doi: 10.1002/ece3.8683 (PMC8901890; doi:10.1002/ece3.8683)
Supplement: Supplementary file 4 — Appendix S4 [file ECE3-12-e8683-s001.docx]

Appendix S4. Additional results of analyses for bacterial communities inside seeds.

Table S4.1. Results of analysis of variance comparing the proportion of each phylum among *C. bursa-pastoris* natural populations. F ratios and their *P* values are given.

| Phylum/class | F | P-value |
| --- | --- | --- |
| Acinobacteria | 3.7188 | 0.0610 |
| Alphaproteobacteria | 3.4804 | 0.0703 |
| Bacteroidetes | 0.3363 | 0.7794 |
| Chloroflexi | 1.000 | 0.4411 |
| Deinococcus | 1.000 | 0.4411 |
| Deltaproteobacteria | 0.6885 | 0.5840 |
| Dependentiae | 1.000 | 0.4411 |
| Firmicutes | 2.9153 | 0.1006 |
| Fusobacteria | 1.000 | 0.4411 |
| Gammaproteobacteria | 1.5943 | 0.2655 |
| Proteobacteria_unclassified | 1.0202 | 0.4333 |

Table S4.2. OTU number and Inverse Simpson index of seed endophytic bacteria.

|  | BAE | DEM | GUM | MOO |
| --- | --- | --- | --- | --- |
| Number of OTUs | 21 | 45 | 20 | 32 |
| Inverse Simpson index | 3.11 (1.38) | 5.47 (1.62) | 6.77 (0.99) | 3.04 (0.61) |
